# Esophageal pressure monitoring and its clinical significance in severe blast lung injury

**DOI:** 10.3389/fbioe.2024.1280679

**Published:** 2024-05-09

**Authors:** Shifeng Shao, Zhengbin Wu, Yi Wang, Yaoli Wang, Zhen Wang, Huan Ye, Hui Zhao

**Affiliations:** ^1^ Department of ICU, Daping Hospital, Army Medical University, Chongqing, China; ^2^ The Fifth Outpatient Clinic, Western Theater General Hospital, Chengdu, China; ^3^ Department of Rehabilitation, The Third People’s Hospital of Chengdu, Chengdu, China; ^4^ Institute for Traffic Medicine, Daping Hospital, Army Medical University, Chongqing, China

**Keywords:** esophageal pressure monitoring, respiratory mechanics, blast lung injury, animal model, biological shock tube

## Abstract

**Background:**

The incidence of blast lung injury (BLI) has been escalating annually due to military conflicts and industrial accidents. Currently, research into these injuries predominantly uses animal models. Despite the availability of various models, there remains a scarcity of studies focused on monitoring respiratory mechanics post-BLI. Consequently, our objective was to develop a model for monitoring esophageal pressure (Pes) following BLI using a biological shock tube (BST), aimed at providing immediate and precise monitoring of respiratory mechanics parameters post-injury.

**Methods:**

Six pigs were subjected to BLI using a BST, during which Pes was monitored. We assessed vital signs; conducted blood gas analysis, hemodynamics evaluations, and lung ultrasound; and measured respiratory mechanics before and after the inflicted injury. Furthermore, the gross anatomy of the lungs 3 h post-injury was examined, and hematoxylin and eosin staining was conducted on the injured lung tissues for further analysis.

**Results:**

The pressure in the experimental section of the BST reached 402.52 ± 17.95 KPa, with a peak pressure duration of 53.22 ± 1.69 ms. All six pigs exhibited an anatomical lung injury score ≥3, and pathology revealed classic signs of severe BLI. Post-injury vital signs showed an increase in HR and SI, along with a decrease in MAP (*p* < 0.05). Blood gas analyses indicated elevated levels of Lac, CO_2_-GAP, A-aDO_2_, HB, and HCT and reduced levels of DO_2_, OI, SaO_2_, and OER (*p* < 0.05). Hemodynamics and lung ultrasonography findings showed increased ELWI, PVPI, SVRI, and lung ultrasonography scores and decreased CI, SVI, GEDI, and ITBI (*p* < 0.05). Analysis of respiratory mechanics revealed increased Ppeak, Pplat, Driving P, MAP, PEF, Ri, lung elastance, MP, Ptp, Ppeak − Pplat, and ΔPes, while Cdyn, Cstat, and time constant were reduced (*p* < 0.05).

**Conclusion:**

We have successfully developed a novel respiratory mechanics monitoring model for severe BLI. This model is reliable, repeatable, stable, effective, and user-friendly. Pes monitoring offers a non-invasive and straightforward alternative to blood gas analysis, facilitating early clinical decision-making. Our animal study lays the groundwork for the early diagnosis and management of severe BLI in clinical settings.

## 1 Introduction

It has been observed that between 17% and 47% of fatalities post-explosion suffer from blast lung injury (BLI), with the prevalence exceeding 90% in terrorist attacks occurring in enclosed spaces such as trains (Katz et al., 1989; Mellor and Cooper, 1989; Arnold et al., 2003; Scott et al., 2017). Furthermore, over 44% of hospitalized patients and 71% of critically ill individuals were found to have lung injuries. Treating severe BLI often necessitates advanced life support, such as mechanical ventilation. However, a specific standard for the mechanical ventilation of patients with primary BLI is lacking, with the current best practices being derived from protocols for the management of acute respiratory distress syndrome (ARDS). The selection of ventilation strategies is varied, and the complexities of BLI intensify the challenge of identifying the most effective ventilation approach. Recent studies suggest that monitoring respiratory mechanics using esophageal pressure (Pes) can offer significant insights for treating respiratory difficulties associated with BLI. Yet, no standardized methodology for ventilatory treatment modalities and parameter settings for severe BLI have been proposed, presenting a considerable challenge to the medical community (Scott et al., 2020).

Currently, both nationally and internationally, there is a paucity of research on the changes in respiratory mechanics following severe BLI. Most animal-based studies have focused on the mechanisms of injury, post-injury pathophysiological alterations, and their underlying molecular mechanisms (Scott et al., 2017; Nguyen et al., 2019; Smith and Garner, 2019; Hazell et al., 2022; Shakargy et al., 2022; Al-Hajj et al., 2023). There has been insufficient exploration into the maintenance programs for post-injury respiratory function, especially those concerning variations in respiratory mechanics parameters monitored by Pes in animals afflicted with severe BLI; such studies are notably absent in the existing literature.

Among the various animal models for BLI, the shock tube represents the most commonly used injury device in laboratories. Consequently, we developed a porcine model of severe BLI under Pes monitoring, employing a large biological shock tube (BST) to induce injury. This study aimed to investigate the alterations in respiratory mechanics parameters following severe BLI in pigs, thereby providing a foundational animal model for subsequent research on respiratory function management strategies post-BLI.

## 2 Materials and methods

### 2.1 Animal preparation

Six Panamanian pigs (aged 6–7 months, all male, weighing on average 27.23 ± 1.84 kg) were sourced from the Animal Experimentation Center of the Army Specialty Medical Center [Animal Production License No. SCXK (Yu) 2017-0002 and Animal Use License No. SYXK (Yu) 2017-0002]. The study was approved by the Ethics Committee for Animal Experimentation of the Army Military Medical University (Ethics Approval No. AMUWE20223478). All animal procedures were conducted following the Guide for the Care and Use of Laboratory Animals.

### 2.2 Instruments and equipment

**FIGURE 1:**
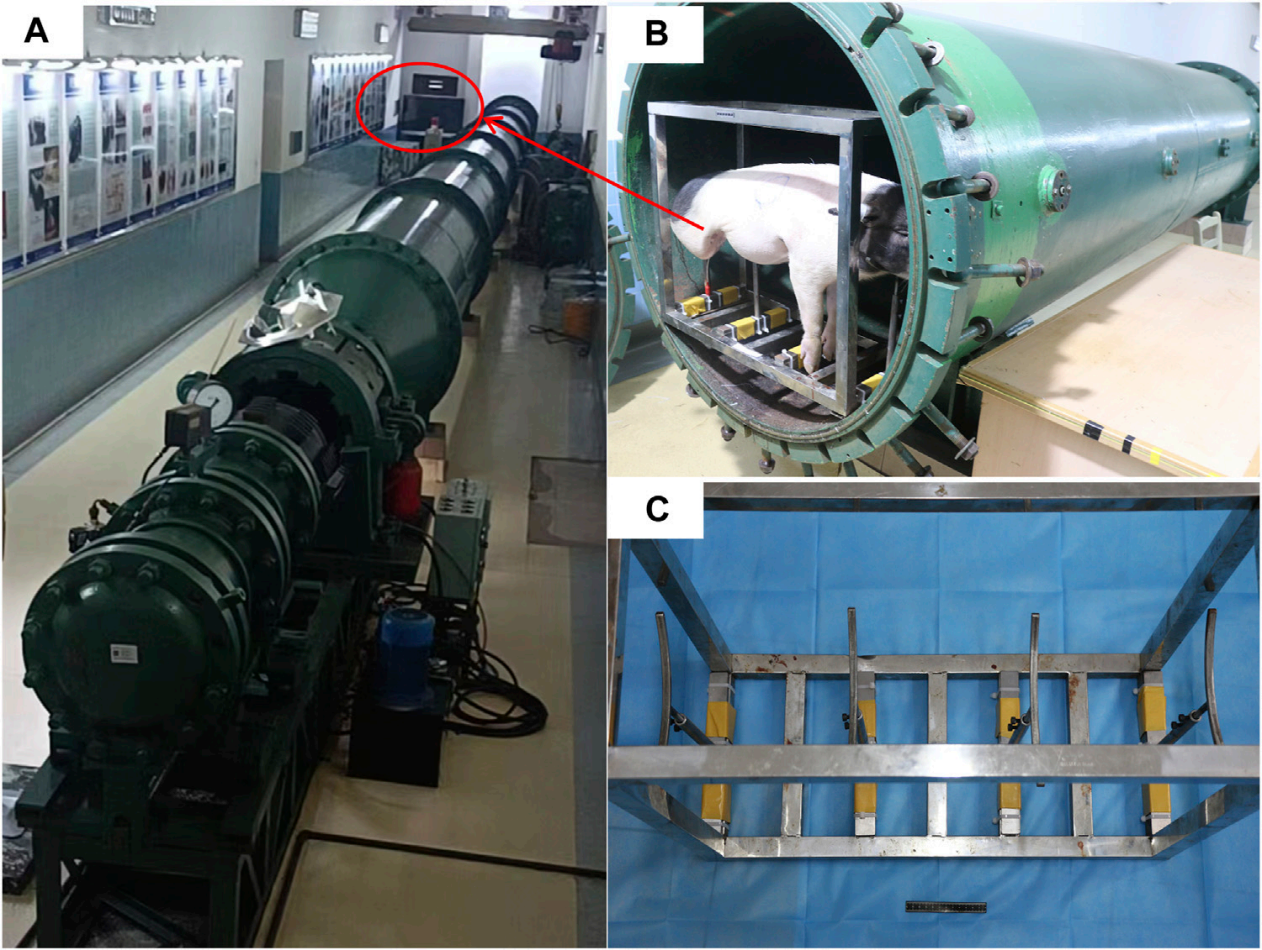
Presentation of the injury device. **(A)** BST-Ⅰ type biological shock tube. **(B)** Animals placed in the experimental section before the injury. **(C)** Animal support auxiliary device.

In this study, we used a BST-I type shock tube based on the compressed air principle (Figure 1). Equipment and materials included a 24G closed venous indwelling needle (Intima, China), a monitor (Mindray, China), a 5-F double-lumen central venous catheter (Medical Components of America, USA), a 4-F PiCCO catheter (Pulsion Medical Systems SE, Germany), a handheld ultrasound device (Huaxi, China), a portable blood gas analyzer (Abbott, USA), and a 3-mL arterial blood collection syringe (BD, England).

### 2.3 Establishment of animal models

**FIGURE 2:**
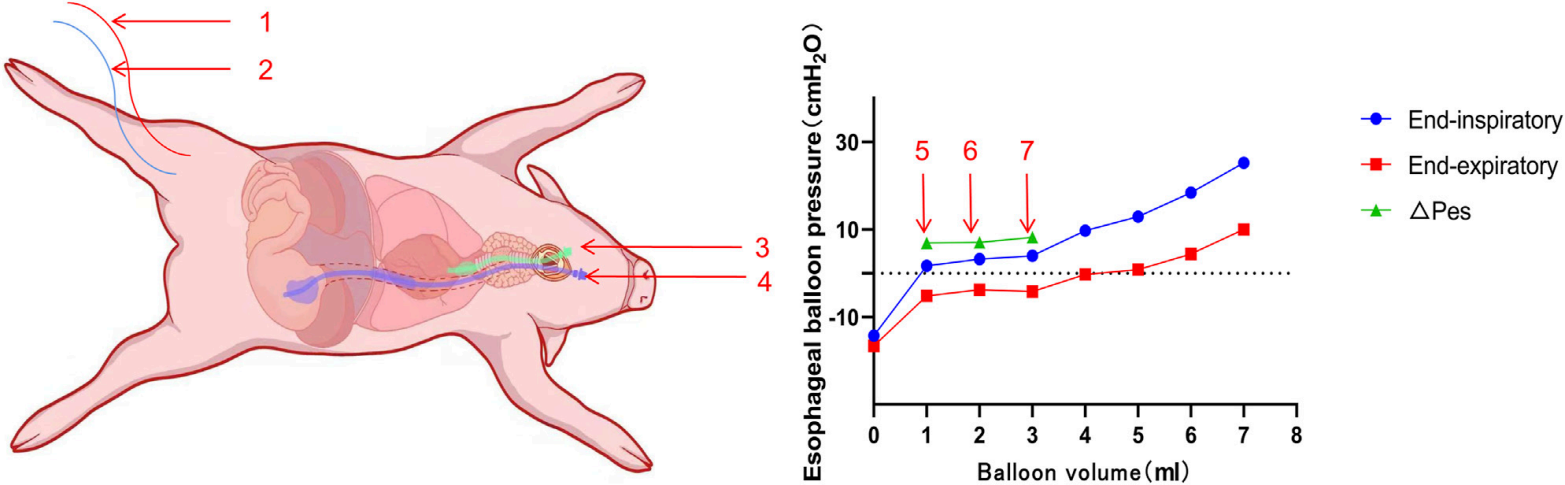
Schematic diagram of the Panamanian pig showing the settings and esophageal balloon pressure–balloon volume curves. 1. Femoral artery catheter. 2. Central venous catheter via the femoral vein. 3. Tracheal intubation tube. 4. Pes catheter. 5. Vmin. 6. Vbest. 7. Vmax.

Before inducing BLI, anesthesia was initiated with a 4 mg/kg intravenous injection of propofol into the ear vein, which was followed by continuous infusion of propofol (3.2–6 mg/kg/h), esketamine (0.4–0.65 mg/kg/h), and fentanyl (0.4–0.65 μg/kg/h) for analgesia and sedation. The depth of sedation was monitored using the bispectral index of the electroencephalogram, aiming for a score of 60–80. Pain levels were assessed using the Critical Care Pain Observation Tool, with a score of 0 indicating no pain. Following the stabilization of anesthesia, the animals were secured in a supine position on the operating table. A neck incision was made for blunt separation to expose the pharynx, followed by an incision above the thyroid cartilage for tracheal intubation. A tracheal intubation cannula (Elmac, China) was inserted through the vocal folds and connected to a ventilator (Padus 8, China) set to volume-controlled ventilation: tidal volume of 300 mL, oxygen concentration of 21%, PEEP of 5 cm H_2_O, inspiratory time of 1 s, and a respiratory rate of 20 breaths per minute. Respiratory mechanics parameters were monitored following the intravenous administration of vecuronium bromide (0.05 mg/kg). A cannula for Pes monitoring (Mindray, China) was inserted through the pharyngeal region. To access the femoral artery and vein, an incision was made in the right lower limb, and the muscle was bluntly separated. A 4-F PiCCO catheter (Pulsion Medical Systems SE, Germany) was inserted into the femoral artery, and a 5-F double-lumen central venous catheter (Medical Components of America, USA) was inserted into the femoral vein using the Seldinger technique (Yu et al., 2022). The tip of the femoral vein catheter was positioned within 2 cm of the right atrium opening, as confirmed by ultrasound (Wisonic, China). Arterial blood pressure and central venous pressure were measured via the femoral artery and venous catheters, respectively. The correct placement of the manometric tube in the stomach was verified by either aspirating the gastric fluid or through auscultation. The transition from intra-abdominal to intrathoracic pressure waveform during gradual catheter withdrawal indicated the balloon’s entry into the esophagus, while a heartbeat artifact on pressure tracing suggested proximity. A ΔPaw/ΔPes ratio between 0.8 and 1.2 confirmed proper localization. If incorrect, the catheter was repositioned and the measurements taken again. A single Panamanian pig was randomly selected to establish the optimal ventilation (Vbest) settings (Figure 2) (Jiang et al., 2022). Subsequently, relevant parameters, such as vital signs, blood gas analysis, lung ultrasound, and respiratory mechanics, were collected.

BLI induction: The animal was positioned in the experimental section of the BST (developed by the Third Affiliated Hospital of Army Medical University, China), supported by a bracket to stand on its left side facing the source of the shock wave. Based on prior studies, a driving pressure of 4.8 MPa was applied to induce BLI. Subsequent to the injury, targeted analgesia and sedation were administered. The overpressure from the experimental shock wave was recorded using a data acquisition system (DH8301N).

Post-BLI assessment: After blast exposure, the same set of parameters as pre-injury parameters was collected to evaluate the effects of the blast injury on the animal. These included vital signs, blood gas analysis, hemodynamics, lung ultrasound, and respiratory mechanics.

General procedure: All procedures were conducted while the animals were under continuous anesthesia to ensure humane treatment and minimize distress.

### 2.4 Collection of relevant parameters for animal models

Following the completion of all animal procedures and a stabilization period of 20 min, analgesia, sedation, and muscle relaxation with rocuronium (5 mL/10 mg) were administered to inhibit spontaneous respiration. Vital signs, such as heart rate (HR), mean arterial pressure (MAP), and SpO_2_ (pulse oxygen saturation), were continuously monitored and automatically recorded using an electrocardiogram monitor.

Blood gas analysis: Arterial blood samples were drawn from the femoral artery catheter, and central venous blood samples were obtained from the femoral vein central venous catheter. These samples were immediately analyzed using a blood gas analyzer (Abbott, USA).

Measurement of hemodynamic parameters: Hemodynamic parameters were assessed using a bolus injection of 10 mL of 0.9% physiological saline (isotonic sodium chloride injection, chilled to 0°C–4°C) administered into the central venous catheter within 7 seconds. Three consecutive measurements were taken, with the average value used for hemodynamic analysis.

#### 2.4.1 Parameter calculation formula

Oxygenation index (OI) = PaO_2_/FiO_2_ ratio.

Pulmonary arterial oxygen tension (PAO_2_) = FiO_2_ × (760 − 47) − PaCO_2_/0.8.

Difference of alveoli − arterial oxygen pressure (A-aDO_2_) = PAO_2_ − PaO_2_.

CO_2_-GAP = PcvCO_2_ − PaCO_2_.

Oxygen extraction ratio (OER) = DO_2_/VO_2_,

DO_2_ (mL/kg/min): DO_2_ = CO × Hb × 1.36 × SaO_2_ + PaO_2_ × 0.0031,

VO_2_ (mL/kg/min): VO_2_ = Hb × 1.34 × SaO_2_ − SvO_2_ × 10 × CO.

Re = Driving P/PEF.

*The placement of the femoral vein catheter tip was accurately localized at the opening of the right atrium using ultrasound guidance. Consequently, mixed venous oxygen saturation (SvO_2_) was substituted with central venous oxygen saturation (ScvO_2_) for this study (Endo et al., 2021).

Lung ultrasonography was conducted according to the BLUE-plus protocol using the ten-zone method. This involved the collection of data from five specified points: upper blue points, lower blue points, diaphragm points, PLAPS points, and posterior blue points on both the left and right sides of the lungs. The lung ultrasonography scoring (cLUSS) criteria were established as follows: score 0 for A-line or ≤2 B-lines; score 1 for ≥3 B-lines; score 2 for diffuse B-lines; and score 3 for tissue-like signs (Mongodi et al., 2017).

Following the administration of analgesia, sedation, and muscle relaxation, respiratory mechanics parameters were measured using a ventilator. The operational procedures and methods were in accordance with those outlined by Yoshida and Brochard (2018) and Jiang et al. (2022). Measurements were taken three times for each parameter, and the average values were used for the analysis.

These parameters were systematically collected both before and after the induction of injury.

### 2.5 Gross and histologic assessment of the extent of lung injury

Three hours post-injury, the animals were euthanized via injection of an overdose of anesthetics, and a necropsy was performed. Pathological features such as pulmonary hemorrhage, lacerations, percentage of hemorrhagic area, and hemorrhagic pleural effusion were documented. The severity of the injuries was assessed using the pathologic severity scale of lung blast injury (PSSLBI), which assigns scores from 1 to 4, corresponding, respectively, to mild, moderate, severe, and extremely severe BLI (Jihong, 2018).

### 2.6 Measurement of the dry and wet weight of lung tissue

The lung tissue, excluding the trachea and main bronchi, was weighed and then dried in an oven at 60°C until a constant weight was achieved. The lung coefficient was calculated: lung coefficient = (lung wet weight/body weight) × 100%. The lung wet/dry weight ratio (W/D) was determined: W/D = lung wet weight/lung dry weight. Additionally, the lung water content was calculated: lung water content = [(lung wet weight − lung dry weight)/lung wet weight] × 100%.

### 2.7 Statistical analysis

The experimental data were analyzed using SPSS version 27.0, Microsoft Excel, and GraphPad Prism 8 software. Normally distributed measurement data were expressed as mean ± standard deviation (SD). Comparisons between groups were performed using one-way repeated measures analysis of variance. Non-normally distributed data were presented as median (25th–75th percentile), and intergroup comparisons were conducted using the Wilcoxon test. Frequencies and percentages were also calculated for categorical data. A *p*-value < 0.05 was considered statistically significant.

## 3 Results

### 3.1 General conditions of animals

**FIGURE 3:**
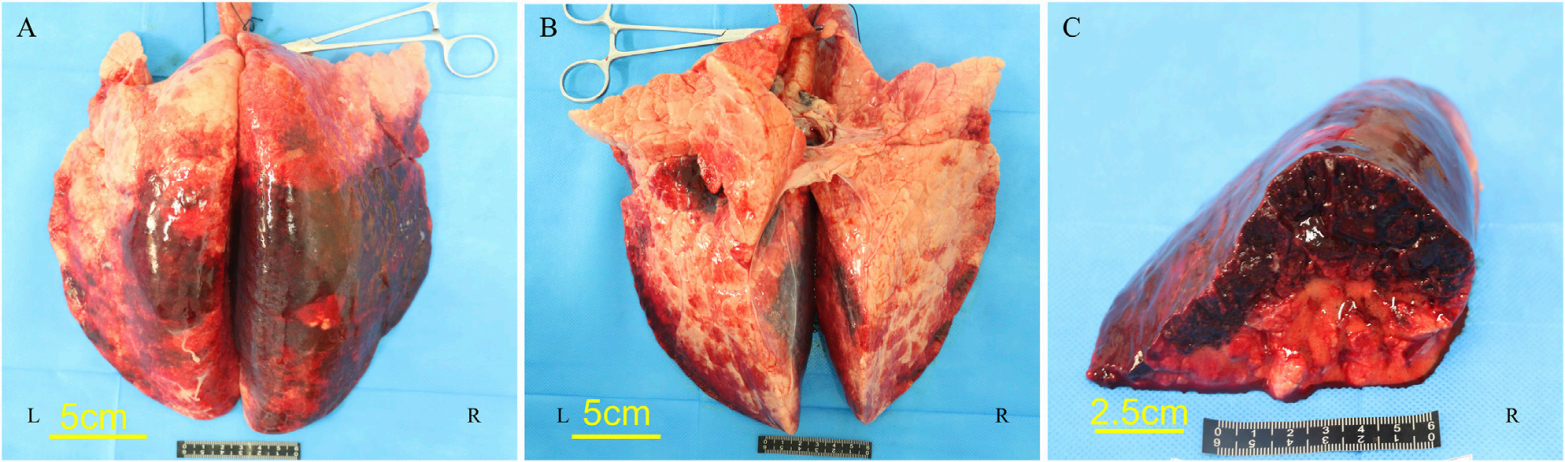
Lung microgram of pig no. 5. **(A)** Dorsal side. **(B)** Ventral side. **(C)** Cross-section of the right lower lobe of the lung.

**FIGURE 4:**
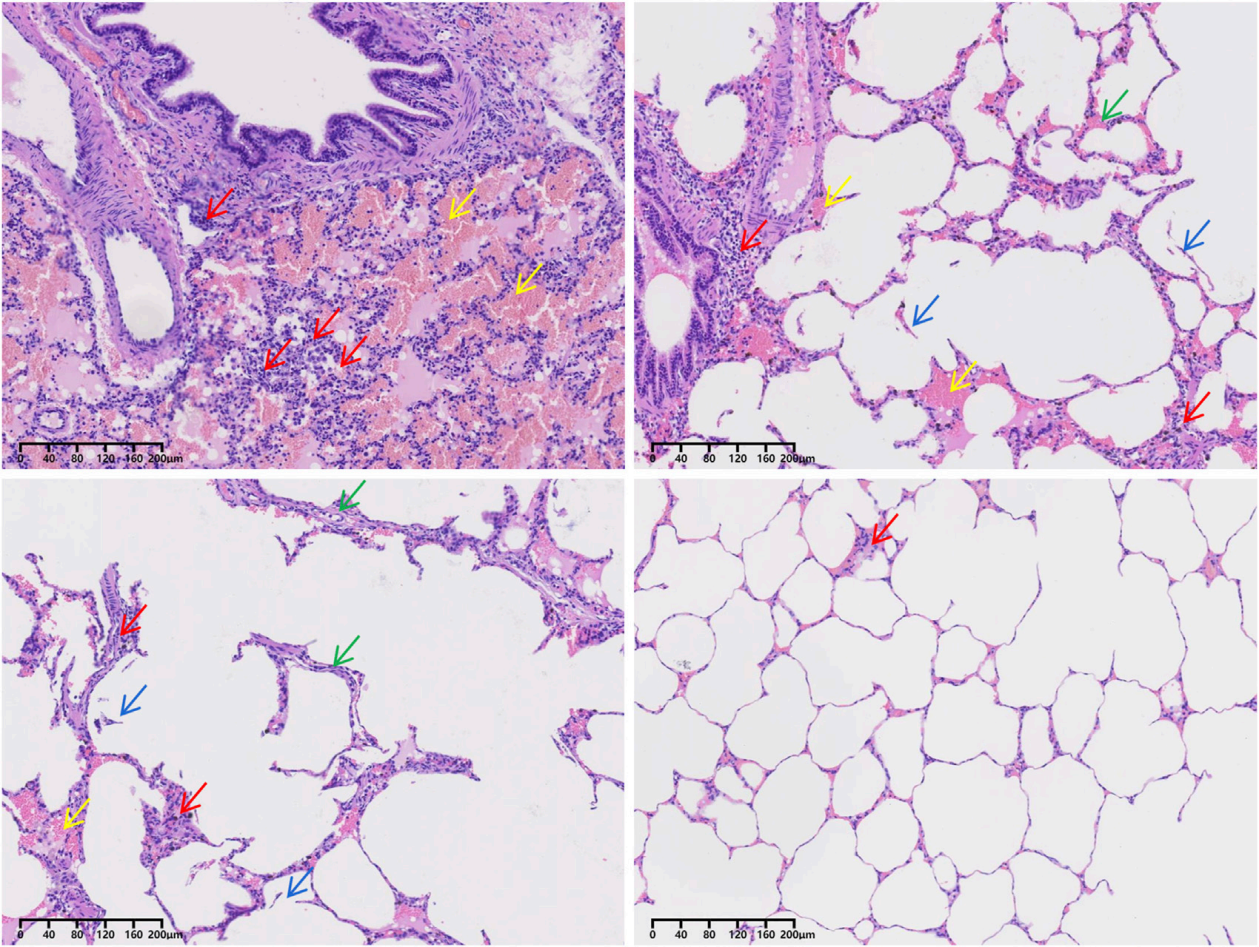
H&E staining of the lung tissue of pig no. 5. Red arrows point to inflammatory cells. Yellow arrows point to red blood cells. Green arrows point to pulmonary interstitial edema. Blue arrows point to alveolar rupture.

**FIGURE 5:**
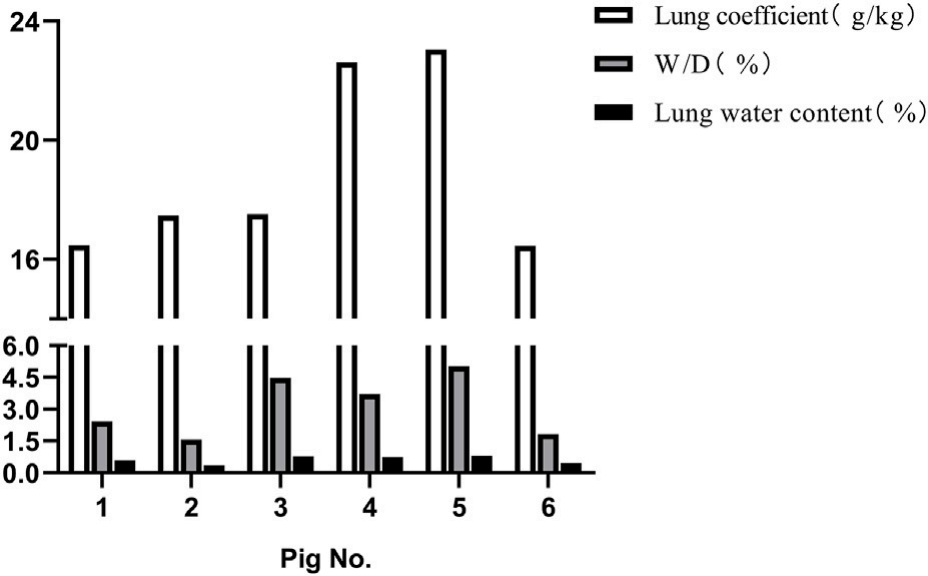
Correlation coefficient of lung water content after blast injury.

Following the injury, immediate assessment was conducted on the animals’ overall condition. There were no visible external injuries on the animals’ body surfaces or apparent fractures; however, all six animals exhibited varying degrees of bloody secretions from the airways. The experimental section of the BST registered a pressure of 402.52 ± 17.95 KPa, with a peak pressure duration of 53.22 ± 1.69 ms. The survival rates dropped to 50% after 1 h and to 16.7% 3 h post-injury. Anatomical evaluations conducted 3 h post-injury revealed that all six animals scored ≥3 points on the PSSLBI (Figure 3), indicating severe injuries. Optical microscopic examination revealed typical BLI features, such as alveolar rupture, intra-alveolar hemorrhage, and inflammatory exudation in the alveolar interstitium (Figure 4). The lung coefficient, W/D ratio, and lung water content, measured in the six animals, were 18.93 ± 3.06, 3.17 ± 1.44, and 61.84% ± 18.15%, respectively (Figure 5), confirming the presence of severe or greater BLI.

### 3.2 Relevant parameters of experimental animal models

**FIGURE 6:**
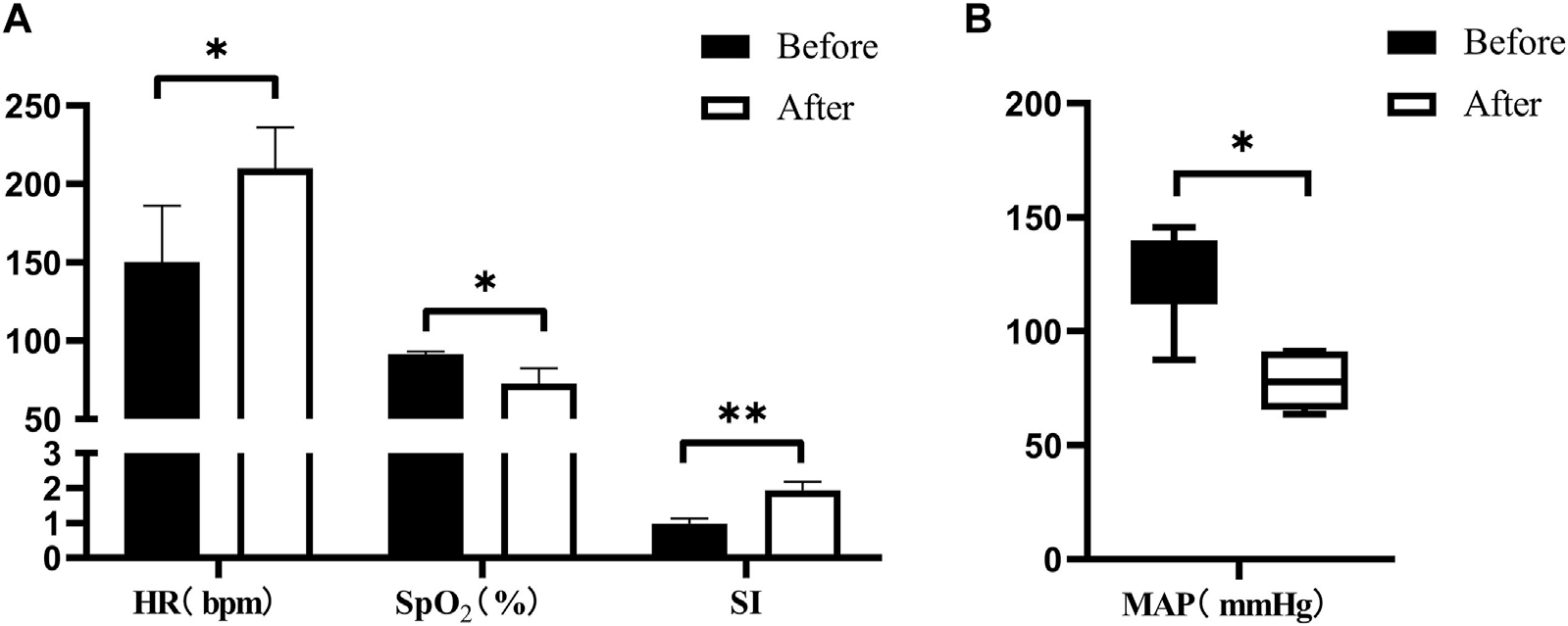
Changes in vital signs after severe BLI. **(A)** Pre- < post-injury HR (RM ANOVA, F = 15.384, *p* = 0.017), pre- > post-injury SpO_2_ (RM ANOVA, F = 20.34, *p* = 0.011), pre- < post-injury SI (RM ANOVA, F = 35.735, *p* = 0.004). **(B)** Pre- > post-injury MAP (Wilcoxon, Z = −2.023, *p* = 0.043). *p < 0.05, **p < 0.01, ***p < 0.001. Before: blast lung before. After: blast lung after.

Post-injury, the animals exhibited significant alterations in vital signs. Following severe BLI, there was an observed increase in HR and SI, whereas MAP and SpO_2_ declined (Figure 6).

**FIGURE 7:**
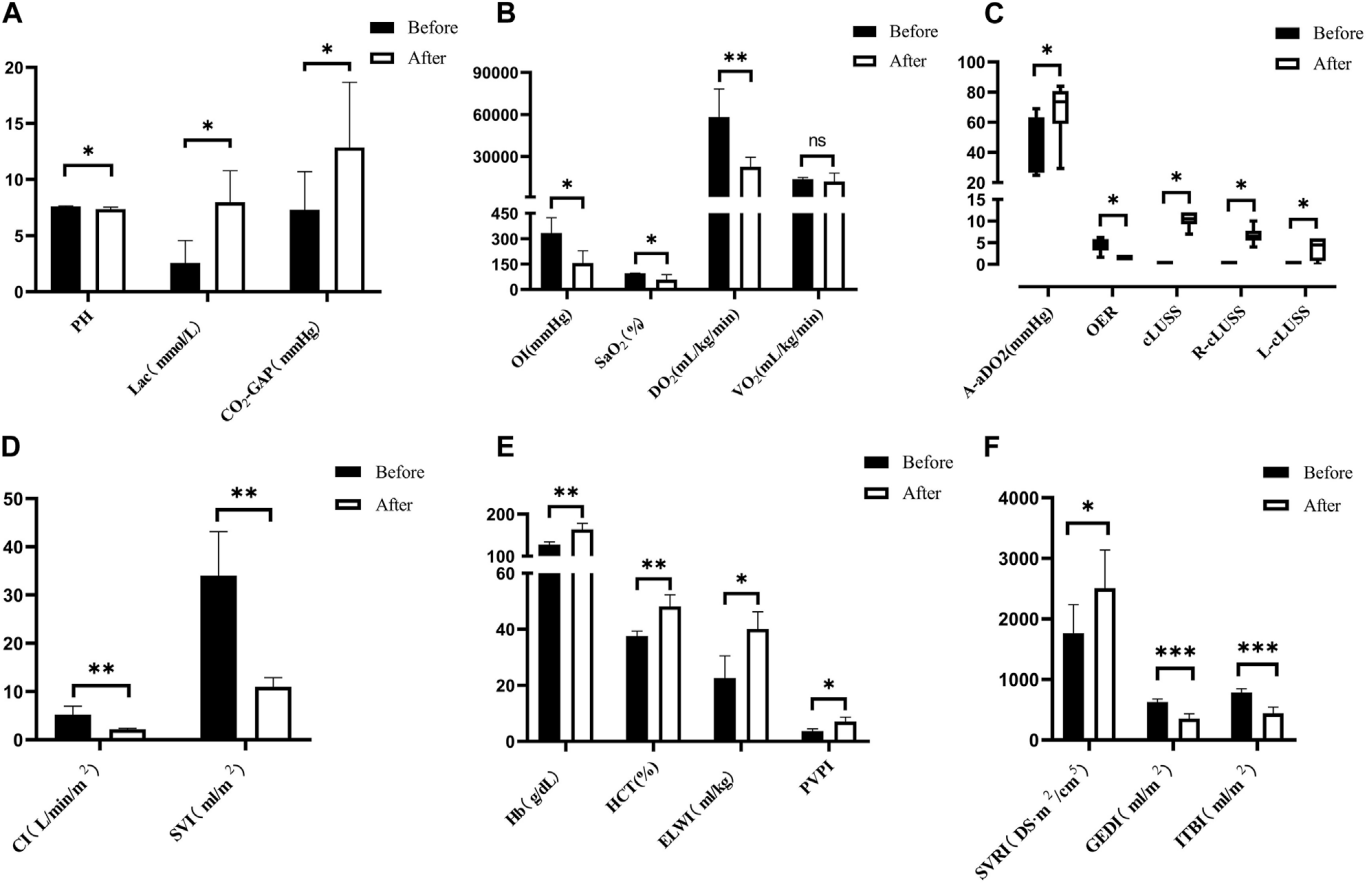
Changes in arterial blood gas analysis for severe BLI. **(A)** Pre- > post-injury PH (RM ANOVA, F = 8.572, *p* = 0.033); pre- < post-injury Lac (RM ANOVA, F = 13.114, *p* = 0.015); pre- < post-injury CO_2_-GAP (RM ANOVA, F = 12.317, *p* = 0.025). **(B)** Pre- > post-injury OI (RM ANOVA, F = 7.764, *p* = 0.039), pre- > post-injury SaO_2_ (RM ANOVA, F = 7.883, *p* = 0.038), pre- > post-injury DO_2_ (RM ANOVA, F = 36.246, *p* = 0.002). There was no statistically significant difference between VO_2_ pre-injury and post-injury (*p* > 0.05). **(C)** Pre- < post-injury A-aDO_2_ (Wilcoxon, Z = −1.992, *p* = 0.046), pre- > post-injury ORE (Wilcoxon, Z = −2.201, *p* = 0.028 < 0.05), pre- < post-injury cLUSS (Wilcoxon, Z = −2.214, *p* = 0.027), pre- < post-injury R-cLUSS (Wilcoxon, Z = −2.214, *p* = 0.027), pre- < post-injury L-cLUSS (Wilcoxon, Z = −2.032, *p* = 0.042). **(D)** Pre- > post-injury CI (RM ANOVA, F = 50.715, *p* = 0.002), pre- > post-injury SVI (RM ANOVA, F = 37.612, *p* = 0.004). **(E)** Pre- < post-injury HB (RM ANOVA, F = 52.155, *p* = 0.010), pre- < post-injury HCT (RM ANOVA, F = 52.245, *p* = 0.010), pre- < post-injury ELWI (RM ANOVA, F = 14.004, *p* = 0.013), pre- < post-injury PVPI (RM ANOVA, F = 10.45, *p* = 0.032). **(F)** Pre- < post-injury SVRI (RM ANOVA, F = 20.132, *p* = 0.011), pre- > post-injury GEDI (RM ANOVA, F = 102.765, *p* < 0.001), pre- > post-injury ITBI (RM ANOVA, F = 104.365, *p* < 0.001). *p < 0.05, **p < 0.01, ***p < 0.001. ns: no significance. Before: blast lung before. After: blast lung after.

Following severe BLI, the blood gas analysis revealed significant physiological changes. There was a notable increase in lactate (Lac), arterial and venous carbon dioxide pressure difference (CO_2_-GAP), alveolar–arterial oxygen pressure difference (A-aDO_2_), hemoglobin (Hb), and hematocrit (HCT). Conversely, there was a decrease in pH, oxygen delivery (DO_2_), oxygenation index (OI), arterial oxygen saturation (SaO_2_), and oxygen extraction ratio (OER), as depicted in Figures 7A–C,E. However, there were no statistically significant differences in transcutaneous–arterial PCO_2_ (Tc-artPCO_2_), the total carbon dioxide content in the plasma (TCPCO_2_), partial pressure of carbon dioxide (PCO_2_), bicarbonate (HCO_3_^−^), end-tidal respiratory carbon dioxide, oxygen consumption (VO_2_), and calcium (Ca^2+^) levels before and after the injury (*p* > 0.05).

Hemodynamic parameters demonstrated significant alterations. The cardiac index (CI), stroke volume index (SVI), global end-diastolic index (GEDI), and intrathoracic blood volume index (ITBI) were significantly decreased (*p* < 0.05), indicating compromised cardiac function and reduced blood volume within the chest cavity. Conversely, there was a significant increase in the systemic vascular resistance index (SVRI), extravascular lung water index (ELWI), and pulmonary vascular permeability index (PVPI) (*p* < 0.05), as shown in Figures 7D–F, reflecting increased vascular resistance and pulmonary edema. However, there was no statistically significant difference in the global ejection fraction (GEF) between pre-injury and post-injury measurements (*p* > 0.05), suggesting that the overall contractility of the heart remained unchanged.

Post-injury, there was a noticeable increase in the lung ultrasound score compared to the pre-injury values, as illustrated in Figure 7C.

**FIGURE 8:**
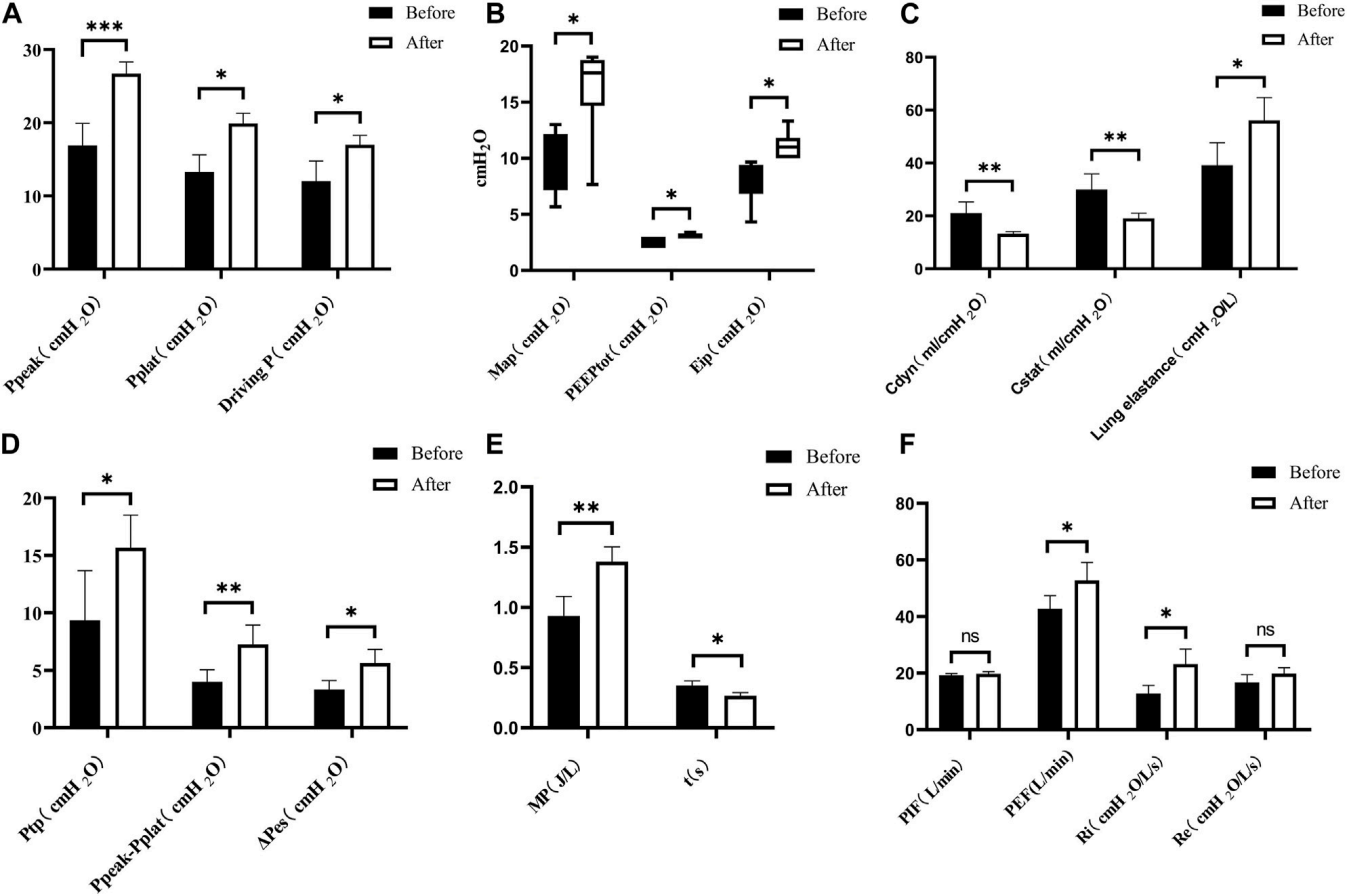
Changes in respiratory mechanics parameters. **(A)** Pre- < post-injury Ppeak (RM ANOVA, F = 69.56, *p* < 0.001), pre- < post-injury Pplat (Wilcoxon, Z = −1890, *p* = 0.021), pre- < post-injury Driving P (RM ANOVA, F = 14.238, *p* = 0.020). **(B)** Pre- < post-injury MAP (Wilcoxon, Z = −2.032, *p* = 0.042), pre- < post-injury PEEPtot (Wilcoxon, Z = −2.060, *p* = 0.039), pre- < post-injury Eip (Wilcoxon, Z = −2.201, *p* = 0.028). **(C)** Pre- > post-injury Cdyn (RM ANOVA, F = 23.389, *p* = 0.008), pre- > post-injury Cstat (RM ANOVA, F = 20.607, *p* = 0.006), pre- < post-injury lung elastance (RM ANOVA, F = 20.892, *p* = 0.010). **(D)** Pre- < post-injury Ptp (RM ANOVA, F = 10.051, *p* = 0.034), Ppeak − Pplat (RM ANOVA, F = 77.076, *p* = 0.003), pre- < post-ΔPes (RM ANOVA, F = 32.772, *p* = 0.011). **(E)** Pre- < post-injury MP (RM ANOVA, F = 22.089, *p* = 0.009), pre- > post-injury time constant t (RM ANOVA, F = 17.228, *p* = 0.014). **(F)** Pre- < post-injury PEF (RM ANOVA, F = 10.682, *p* = 0.031), pre- < post-injury Ri (RM ANOVA, F = 39.113, *p* = 0.003), no statistically significant difference in PIF (peak inspiratory flow) and Re (expiratory resistance) (*p* > 0.05). *p < 0.05, **p < 0.01, ***p < 0.001. ns: no significance. Before: blast lung before. After: blast lung after.

Post-severe BLI, there were significant changes in the respiratory mechanics observed in the pigs. Parameters such as peak inspiratory pressure (Ppeak), end-inspiratory plateau airway pressure (Pplat), driving pressure (Driving P), esophageal end-inspiratory pressure (Eip), mean airway pressure (MAP), transpulmonary pressure (Ptp), peak expiratory flow (PEF), inspiratory resistance (Ri), mechanical power (MP), lung elasticity, the difference between peak pressure and plateau pressure (Ppeak − Pplateau), and esophageal oscillatory pressure (ΔPes) all exhibited increases. By contrast, dynamic lung compliance (Cdyn), static compliance (Cstat), and time constant showed decreases, as depicted in Figure 8. There were no statistically significant differences in total positive end-expiratory pressure (total PEEP), intrinsic PEEP (PEEPi), peak inspiratory flow (PIF), and expiratory resistance (Re) post-injury (*p* > 0.05).

## 4 Discussion

BLI represents one of the most prevalent types of injuries in warfare and chemical manufacturing explosions; however, these injuries are often insidious and challenging to detect (Al-Hajj et al., 2021; Tabakan et al., 2021). Accurate modeling and early identification of the evolution of blast injuries are imperative for both scientific research and clinical management of BLI. Blast injuries typically occur abruptly, rendering prevention difficult. Consequently, early detection and intervention in BLI are essential in mitigating the risk of mortality and long-term disability.

Current research on BLI predominantly uses live ammunition or laboratory shock tubes to develop animal models. However, these studies are mainly confined to biomechanics, pathology, and anatomy, with a notable gap in the systematic examination of changes in respiratory mechanics parameters associated with BLI. Efforts are ongoing to identify early detection techniques and strategies for maintaining respiratory function post-injury. Unlike traditional acute lung injury models developed through methods such as intravenous injection of endotoxin, intratracheal instillation of oleic acid, exposure to high oxygen levels, or ventilator-induced injury (Champion et al., 2009; Matute-Bello et al., 2011; Hazell et al., 2022; Rozenfeld et al., 2022), blast injury replicates acute blunt traumatic lung injury caused by external forces impacting the lungs. This mode of injury differs significantly in its mechanism and impact from previous models, which failed to accurately replicate the nuances of acute traumatic blunt lung injuries, particularly lung contusions. BLI represents a distinct category of acute lung injury that does not compromise the structural integrity of the chest wall. Hence, the compliance of the chest wall remains largely unchanged. The primary damage is inflicted within the intrathoracic lungs. Additionally, clinical injury grading standards for BLI differ from those established for ARDS (Pizov et al., 1999; Matthay et al., 2024). The explosion’s shock wave exerts overpressure, dynamic pressure, and other effects on the chest, causing injuries through complex multidimensional forces such as implosion, spallation, and inertia across tissues of varying densities. This results in heterogeneous ruptures of alveolar capillaries, intrapulmonary bleeding, and edema (Wolf et al., 2009; Smith and Garner, 2019).

In this study, Panamanian pig was selected as the experimental subject due to its anatomical and physiological resemblances to humans. The injury was induced using a BST within the laboratory setting, where the shock tube’s driving pressure was meticulously controlled to reliably establish a severe BLI model. To mitigate potential impacts on cerebral blood flow, femoral vein central venous cannulation was employed instead of vascular puncture of the neck. Additionally, the Pes monitoring kit enters the esophagus via the hypopharynx, while tracheal intubation enters the airway through the glottis. The results from this investigation aim to provide a foundation for monitoring of the respiratory mechanics in severe BLI within clinical environments.

Post-injury vital signs indicated an increased HR, a decreased MAP, and an increased SI, aligning with the changes observed in central hemodynamic parameters (CI and SVI), volumetric parameters (GEDI and ITBI), and vascular peripheral resistance (SVRI). These findings are consistent with the lung ultrasonography scores and anatomical observations, corroborating previous clinical retrospective studies and laboratory research on blast injuries (Zhang et al., 2015; Yuanbo et al., 2016; Tong et al., 2018; Smith and Garner, 2019; April et al., 2021; Carius et al., 2022; Al-Hajj et al., 2023). Previous studies have demonstrated that prompt and effective arterial blood gas analysis following BLI is crucial for diagnosing conditions and developing treatment plans (Manera et al., 2020; Xue et al., 2020; Chong et al., 2021; Shi et al., 2022). However, early-stage conditions may lack the necessary means for timely, continuous, and effective arterial blood gas monitoring. Moreover, initiation of artificial airways and ventilator-assisted breathing is essential to ensure adequate oxygenation early in severe BLI cases (Anonymous, 2022). Compared to blood gas analysis, Pes monitoring is simpler to perform and less sensitive to environmental factors such as temperature and atmospheric pressure. Therefore, in severe BLI, Pes monitoring is more feasible, reliable, and stable for early-stage clinical decision-making, potentially supplanting the role of blood gas analysis (Loring et al., 2010; Mauri et al., 2016; Umbrello and Chiumello, 2018; Yoshida and Brochard, 2018; Dostal and Dostalova, 2023). Following severe BLI, both dynamic and static lung compliances decreased, necessitating increased driving pressure for ventilator-assisted breathing to achieve pre-injury tidal volumes. The increase in lung ultrasound score, decrease in blood gas analysis oxygenation index, increase in pulmonary vascular permeability as measured by PiCCO, and increase in lung water content indirectly confirmed the reasons for reduced lung compliance. The fundamental causes were diffuse alveolar bleeding, interstitial inflammatory exudation, and edema. Although bloody secretions increased in the airway post-injury, the variations in airway resistance and peak airway flow rate did not consistently align with the changes in peak and plateau airway pressures, suggesting that bloody secretions are not the primary factor affecting airway resistance changes. This hypothesis is supported by the blood gas analysis showing differences in intra-alveolar partial pressures of oxygen, indicating unique respiratory mechanics changes. While airway pressure measurements provide information about lung ventilatory capacity, they do not fully capture the extent of blast lung damage. By contrast, Pes reflects pleural pressures surrounding the lungs, and monitoring Pes can help better assess lung pressure and stress states. Combining airway pressure and Pes measurements offers a more comprehensive evaluation of the extent of lung damage. Pes testing also indicated that thoracic compliance remained unchanged, and there was no increase in abdominal pressure post-injury. These characteristic changes in respiratory mechanics can accurately guide clinical treatment for severe BLI, minimize unnecessary ventilator-related injuries, and offer insights similar to those observed in ARDS cases (Talmor et al., 2008; Yoshida and Brochard, 2018; Pelosi et al., 2021). Monitoring respiratory mechanics under Pes provides a reliable basis for early-stage clinical decision-making, enabling accurate and personalized respiratory treatments to reduce ventilator-induced lung injuries and guide the entire course of mechanical ventilation in severe BLI cases.

Furthermore, this study employed a large biological shock tube to induce injuries, resulting in stable injury parameters. Unlike previous BLI models that relied on lung ultrasound, blood gas analysis, and imaging for evaluation, monitoring respiratory mechanical changes after BLI through Pes enables more direct and precise quantification of mechanical properties such as airway resistance and lung compliance. The research data can guide clinical practice and provide an experimental foundation (Li et al., 2020; Xue et al., 2020; Yang et al., 2020; Ding et al., 2022). Future research should aim to establish a graded animal model of primary BLI to improve the understanding of the temporal and quantitative relationships between respiratory mechanics and lung injury. This approach could lead to the development of a triage tool to boost the rate of early intervention.

Our study has several limitations, which we plan to address in future research. First, the small sample size could increase the risk of Class 1 errors. Second, to prevent rupture from shock wave impact in the Pes monitoring model, we deflated the balloon during injury to avoid bursting of the Pes and tracheal intubation balloons, necessitating catheter replacement. Lastly, our research was limited to animals with severe or fatal BLI. In the future, we aim to enlarge the sample size, extend the observation period, and include BLI animals with varying levels of injury. Concurrently, we plan to integrate other physiological parameters with respiratory mechanics measurements to establish correlations that aid in early injury detection and guide clinical management.

## Figures and Tables

**Table:** 

**HR**	heart rate
**MAP**	mean arterial pressure
**SpO**_**2**_	percutaneous oxygen saturation
**SI**	shock index
**A-aDO**_**2**_	alveolar oxygen pressure difference
**BLI**	blast lung injury
**BST**	biological shock tube
**Cdyn**	dynamic lung compliance
**CI**	cardiac index
**CO**_**2**_ **-GAP**	arterial and venous carbon dioxide pressure difference
**Cstat**	static compliance
**DO**_**2**_	oxygen delivery
**Driving P**	driving pressure
**ELWI**	extravascular lung water index
**GEDI**	global end diastolic index
**HB**	hemoglobin
**HCT**	Hematocrit
**ITBI**	intrathoracic blood volume index
**Lac**	Lactic
**Map**	mean airway pressure
**MP**	mechanical power
**OER**	oxygen extraction ratio
**OI**	oxygenation index
**PEF**	peak expiratory flow
**Pes**	esophageal pressure
**Ppeak**	peak inspiratory pressure
**Ppeak** − **Pplat**	peak pressure–plateau pressure difference
**Pplat**	plat end-inspiratory plateau airway pressure
**Ptp**	transpulmonary pressure
**PVPI**	pulmonary vascular permeability index
**Ri**	inspiratory resistance
**SaO**_**2**_	arterial oxygen saturation
**SVI**	stroke volume index
**SVRI**	somatic vascular resistance index

## Data Availability

The original contributions presented in the study are included in the article/Supplementary Material; further inquiries can be directed to the corresponding authors.
