# Supplementary material for: Esophageal pressure monitoring and its clinical significance in severe blast lung injury
Source: Front Bioeng Biotechnol. 2024 May 9;12:1280679. doi: 10.3389/fbioe.2024.1280679 (PMC11112033; doi:10.3389/fbioe.2024.1280679)
Supplement: Supplementary file 1 [file Table1.docx]

Supplement table 1. Respiratory mechanics parameters

|  | Before | After | F/Z | P |
| --- | --- | --- | --- | --- |
| Ppeak（cmH_2_O） | 16.92±3.03 | 26.72±1.58 | 69.56 | ＜0.001 |
| Pplat（cmH_2_O） | 13.29±2.35 | 19.92±1.39 | 20.078 | 0.021 |
| Driving P（cmH_2_O） | 12±2.8 | 16.98±1.31 | 14.238 | 0.02 |
| Map（cmH_2_O） | 10（8.67，11.33） | 17.6（17.05，18.5） | -2.032 | 0.042 |
| PEEP（cmH_2_O） | 2.17（2.2.83） | 3（3，3.3） | -2.06 | 0.039 |
| Eip（cmH_2_O） | 9（8,9.25） | 11（10.25,11.25） | -2.201 | 0.028 |
| Ptp（cmH_2_O） | 9.37±4.31 | 15.67±2.84 | 10.051 | 0.034 |
| Ppeak-Pplat（cmH_2_O） | 4±2.11 | 7.28±3.33 | 77.076 | 0.003 |
| ΔPes（cmH_2_O） | 3.3±0.8 | 5.64±0.71 | 32.772 | 0.011 |
| PEF（L/min） | 42.73±4.64 | 52.76±6.4 | 10.982 | 0.031 |
| Ri（cmH_2_O/L/s） | 12.77±2.91 | 23.25±5.32 | 39.113 | 0.003 |
| Cdyn（ml/cmH_2_O） | 21.03±4.25 | 13.26±0.87 | 23.389 | 0.008 |
| Cstat（ml/cmH_2_O） | 29.97±5.94 | 19.11±1.93 | 20.607 | 0.006 |
| lung elastance（cmH_2_O/L） | 39.14±8.54 | 56.11±8.67 | 20.892 | 0.01 |
| MP（J/L） | 0.93±0.16 | 1.38±0.12 | 22.089 | 0.009 |
| t（s） | 0.35±0.04 | 0.27±0.03 | 17.228 | 0.014 |
